# Towards Understanding of Polymorphism of the G-rich Region of Human Papillomavirus Type 52

**DOI:** 10.3390/molecules24071294

**Published:** 2019-04-02

**Authors:** Maja Marušič, Janez Plavec

**Affiliations:** 1 Slovenian NMR Center, National Institute of Chemistry, Hajdrihova 19, SI-1000 Ljubljana, Slovenia; marusic.maja@ki.si; 2 EN-FIST Center of Excellence, SI-1000 Ljubljana, Slovenia; 3 Faculty of Chemistry and Chemical Technology, University of Ljubljana, SI-1000 Ljubljana, Slovenia

**Keywords:** G-quadruplex, NMR, folding, DNA, structure, human papillomaviruses

## Abstract

The potential to affect gene expression via G-quadruplex stabilization has been extended to all domains of life, including viruses. Here, we investigate the polymorphism and structures of G-quadruplexes of the human papillomavirus type 52 with UV, CD and NMR spectroscopy and gel electrophoresis. We show that oligonucleotide with five G-tracts folds into several structures and that naturally occurring single nucleotide polymorphisms (SNPs) have profound effects on the structural polymorphism in the context of G-quadruplex forming propensity, conformational heterogeneity and folding stability. With help of SNP analysis, we were able to select one of the predominant forms, formed by G-rich sequence d(G_3_TAG_3_CAG_4_ACACAG_3_T). This oligonucleotide termed HPV52_(1–4)_ adopts a three G-quartet snap back (3 + 1) type scaffold with four *syn* guanine residues, two edgewise loops spanning the same groove, a no-residue V loop and a propeller type loop. The first guanine residue is incorporated in the central G-quartet and all four-guanine residues from G4 stretch are included in the three quartet G-quadruplex core. Modification studies identified several structural elements that are important for stabilization of the described G-quadruplex fold. Our results expand set of G-rich targets in viral genomes and address the fundamental questions regarding folding of G-rich sequences.

## 1. Introduction

Human papillomaviruses (HPV) are pathogens infecting skin and mucosa that have co-evolved with human species and are therefore well adapted to cause infection with minimal damage to their host. Even though there are currently more than 200 different types of HPVs described [1], only a fraction of those are responsible for the development of diseases in humans. Their life-cycle unravels in synchrony with differentiation of keratinocytes, starting from increased copy number of viral episome in the basal layer, through production of viral protein and finally assembly of viral particles that are shed from mature keratinocytes when they die [2]. In most cases the infection passes unnoticed and is quickly resolved by immune system [3]. A subgroup of HPVs designated as ‘high-risk’ are more potent in driving differentiating keratinocytes into the unscheduled and therefore potentially erroneous cell cycle due to a wider range of binding partners of main viral oncoproteins E6 and E7 [4,5,6]. Consequently, persistent infection with high-risk HPVs can eventually lead to development of neoplasms and even cancer, most commonly cancer of skin, head and neck and anogenital regions [7,8]. Among most potent high-risk HPVs are HPV16, HPV18, HPV52 and HPV58 with different regional distribution around the world and ability to cause disease [9]. 

We have recently examined ability of G-rich sequences in several high-risk HPV types to fold into G-quadruplexes [10], four stranded DNA structures with square planar arrangements of guanine residues [11] stacked on each other and stabilized with cations. Stabilization of G-quadruplexes in cells was shown to induce breaks in double-stranded DNA and lead to genome instability [12,13,14], while reporter assays in different expression systems confirmed effect on protein expression for a wide range of G-rich sequences [15]. Analogously, several G-rich sequences with G-quadruplex forming potential were identified in viruses [16,17,18] and stabilization of G-quadruplexes with ligands could have potential effect on expression and/or stability of viral DNA [19,20,21,22,23,24], especially in viruses with double stranded DNA genomes, such as HPV. However, designing ligands that would specifically bind to G-quadruplexes and lock their structure is hampered due to a large number of potential G-quadruplex-forming sequences in the human genome [25,26,27,28], highlighting the importance of structural characterization of G-rich oligonucleotides to guide ligand design. Most promising G-quadruplex targets possess specific structural features found in loops that could promote specific binding in tandem with large planar surface of G-quartets [29]. Additionally, as G-rich oligonucleotides are notoriously polymorphic, their structure can change with variations in solution conditions (pH, T, oligonucleotide and cation concentrations) and intrinsic attributes of G-rich sequence (length of G-tracts, type of connecting loop residues and 5′- and/or 3′-end flanking residues) [30,31,32]. Selective stabilization of predominant fold and its characterization therefore still represent the main obstacle on the way to design structurally discriminating ligands. Moreover, point mutations or single nucleotide polymorphisms (SNPs) inevitably present in G-rich sequences from natural sources might drastically affect structure and therefore cannot be neglected when assessing structural polymorphism of a given G-rich oligonucleotide. 

**Figure 1:**
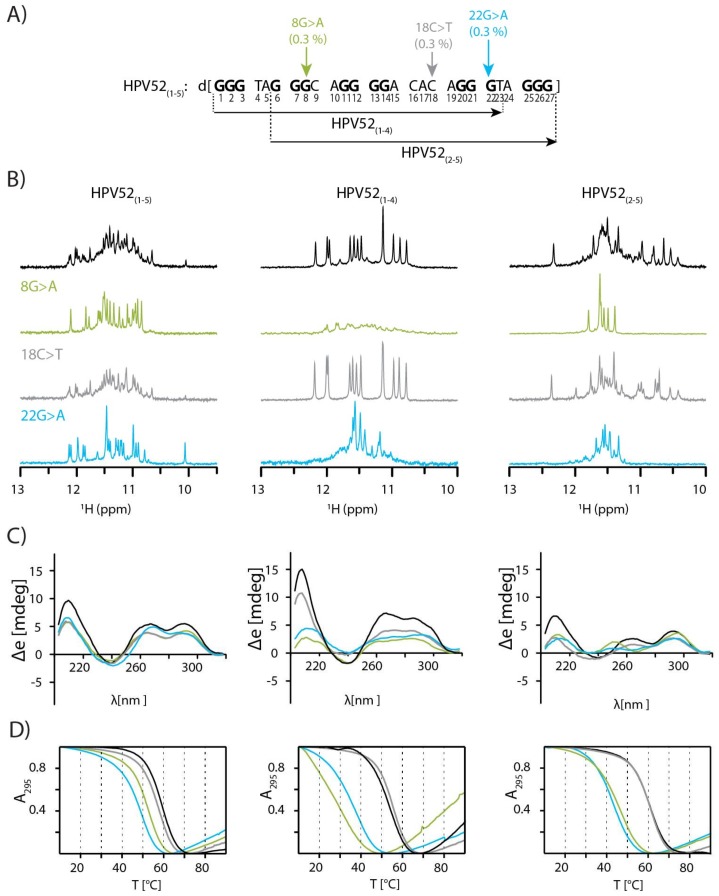
Characterization of G-rich oligonucleotides HPV52_(1–5)_, HPV52_(1–4)_ and HPV52_(2–5)_. (**A**) G-rich sequence of HPV type 52 with indicated position and frequency (in %) of occurrence of observed SNPs in the sequence. (**B**) NMR, (**C**) CD spectra and (**D**) UV melting curves of twelve different oligonucleotides derived from G-rich sequence of HPV type 52. Spectra were recorded at 25 °C, pH 7, 50 mM [K^+^] and c_oligo_ between 0.26 and 0.41 mM (NMR) and 10 µM (CD, UV).

In the current study, we have concentrated our efforts on structure determination of the predominant form of G-rich oligonucleotide from HPV type 52, as well as the characterization of other folds that might exist in dynamic equilibrium and may also be important in the cellular environment. HPV 52 is one of the most relevant HPV types especially in (Southeast) Asia, where it causes up to 20% of all cervical cancer [33]. G-rich oligonucleotide found in the genome of HPV52 forms several different structures due to its five G-rich tracts of different lengths (3, 3, 4, 3, and 3 nt, Figure 1A) [10]. Shortening of the G-rich sequence to four G-tracts was considered as the first approach to reduce polymorphism and be able to identify predominant form(s). By this approach we obtained two shorter oligonucleotides that comprise first to the fourth and the second to the fifth G-tract. The second approach utilizes introduction of point mutations, which can drastically affect number and type of G-quadruplex structure(s). Specifically, three SNPs were found in the genomes of different HPV type 52 isolates in GenBank [34] (Figure 1A) that can be exploited to study effect of nucleotide changes without introduction of artificial sequence changes. SNPs in the G-rich tracts (8G > A and 22G > A) were expected to substantially reduce number of structures in solution, while SNP in loop region (18C > T) was expected to have minimal effect. Selection of the predominant species and its characterization followed by determination of 3D structure to the level of atomic resolution offers a rich collection of NMR parameters uncovering structural elements that are important for stabilization of described G-quadruplex fold and for their recognition by cellular partners or ligands. With introduction of modifications we were able to assess the importance of several structural elements, particularly a four-guanine tract adopting a V loop and a GNA type loop that have been well studied in the context of nucleic acid’s structure and can potentially drive formation of G-quadruplex structure.

## 2. Results

### 2.1. SNPs Reduce Polymorphism of HPV52_(1–5)_ and Assist Identification of Predominant Species

Twenty seven (27) nt long oligonucleotide originating from the genome of HPV type 52 with its five G-rich tracts was expected to adopt a large number of structures, thus posing a challenge for structural studies. Indeed, after titration with aqueous solution of KCl a high number of signals of different intensities was observed in the region between δ 10.0 and 12.2 ppm in the ^1^H-NMR spectrum of HPV52(1–5) (Figure 1B), which indicated its folding into several G-quadruplex structures. Comparison of NMR and CD spectra of HPV52_(1–5)_ without and with SNPs enabled several interesting observations (Figure 1B–D). Spectra of HPV52_(1–5)_ and HPV52_(1–5)_ 18C > T are very similar, which suggests that SNP 18C > T in the third loop does not affect structures formed by the HPV52_(1–5)_ or reduce their number. In contrast, for HPV52_(1–5)_ 8G > A and HPV52_(1–5)_ 22G > A the number of ^1^H-NMR signals in imino region decreases to 24, suggesting reduction of a number of G-quadruplex structures to two in each case. PAGE gel for all four oligonucleotides shows fast moving bands, which are suggestive of formation of monomeric species (Figure S1 and [30]).

From the analysis of the fingerprint imino region it can be inferred that most likely four different structures present for HPV52_(1–5)_ 8G > A and HPV52_(1–5)_ 22G > A are also observed for HPV52_(1–5)_. Especially one of the structures adopted by HPV52_(1–5)_ 22G > A can be easily distinguished due to its upfield signal at δ 10.09 ppm (Figure 1B). Apart from the four structures observed for oligonucleotides with SNPs 8G > A and 22G > A, detailed analysis of imino region of 1D ^1^H spectra revealed at least one additional species for HPV52_(1–5)_. Four structures of HPV52_(1–5)_ that are present for oligonucleotides with SNPs 8G > A and 22G > A most likely incorporate one of the central G-tracts in their loop regions, which results in structures with very long loops (i.e., either 7 or 11 nt). Respective thermal stabilities support this assumption, as apparent T_m_ values decrease for oligonucleotides with SNPs (Figure 1D and Figure S2). Clearly, melting temperature can be determined only for a two-state equilibrium, which is not the case for HPV52_(1–5)_. However, apparent melting temperatures can still give a useful estimation of thermal stability of structures adopted by oligonucleotides and are used as such in our case. The highest apparent T_m_ decrease of 10 °C is observed for HPV52_(1–5)_ 22G > A, which exhibits the longest possible loop (11 nt) within HPV52 G-rich sequence that encompasses the fourth G-tract (Figure 1A). HPV52_(1–5)_, however, displays the highest apparent melting temperature, which allows for several inferences to be made. First, the most stable structure of HPV52_(1–5)_ is not one of those observed for HPV52_(1–5)_ 8G > A and HPV52_(1–5)_ 22G > A since apparent melting temperature for HPV52_(1–5)_ is the highest among the three oligonucleotides. As a consequence, HPV52_(1–5)_ must contain both the second and the fourth G-tracts, which are not present in the structures of HPV52_(1–5)_ 8G > A and HPV52_(1–5)_ 22G > A. Second, the most stable structure of HPV52_(1–5)_ has loops that are shorter than 7 nt, as structures with longer loops are expected to exhibit lower thermal stability [35], as has been observed for HPV52_(1–5)_ 8G > A and HPV52_(1–5)_ 22G > A. Therefore, the most stable structure must also comprise the third G-tract of HPV52_(1–5)_ as a part of the G-quadruplex core. Third, thermally most stable structure is arguably the most relevant in biological context. These initial results suggested that reducing the number of G-tracts at either 5′ or 3′ end might be a favourable strategy for reducing polymorphism of HPV52_(1–5)_, thus enabling determination of topology of the most stable and biologically relevant G-quadruplex structure. Further studies were therefore performed on shorter oligonucleotides, HPV52_(1–4)_ and HPV52_(2–5)_ that comprise the first to the fourth and the second to the fifth G-tract, respectively.

Titration of HPV52_(1–4)_ and HPV52_(1–4)_ 18C > T with aqueous solution of KCl resulted in twelve sharp signals in the region between δ 11.8 and 12.3 ppm, characteristic of a structure with three G-quartets. Position of signals in CD spectra is typical for a (3 + 1) topology (Figure 1B–C) [36]. SNPs 8G > A and 22G > A have detrimental effect on the formation of G-quadruplex structure, more so for the former (Figure 1B). In agreement, a larger decrease in apparent T_m_ was observed for HPV52_(1–4)_ 8G > A (25 °C) than for HPV52_(1–4)_ 22G > A (18 °C) compared to the apparent Tm of parent oligonucleotide HPV52_(1–4)_ (Figure 1D). PAGE gel shows bands with slow(er) mobilities for HPV52_(1–4)_ 8G > A and 22G > A, which are characteristic for formation of intermolecular species, presumably dimers (Figure S1). 

Upon titration of HPV52_(2–5)_ and HPV52_(2–5)_ 18C > T with KCl solution several structures were formed (Figure 1B). CD spectra are characteristic of a (3 + 1) topology, while the apparent T_m_ is 60 °C for both oligonucleotides (Figure 1B–C). For HPV52_(2–5)_ 8G > A eight partially overlapped signals are observed in the region from δ 11.4 and 11.8 ppm of ^1^H-NMR spectrum, which in combination with distribution of CD signals points to the formation of an antiparallel structure with two G-quartets [36]. Similarly, for HPV52_(2–5)_ 22G > A eight partially overlapped signals assigned to antiparallel fold are observed in the imino region of the ^1^H-NMR spectrum. Several other signals of low intensity most likely correspond to non-antiparallel species, since CD spectrum of HPV52_(2–5)_ 22G > A that represents a sum of CD spectra of all species exhibits characteristics for mixture of different topologies [37]. 

### 2.2. Topology of G-quadruplex Adopted by HPV52_(1–4)_

**Figure 2:**
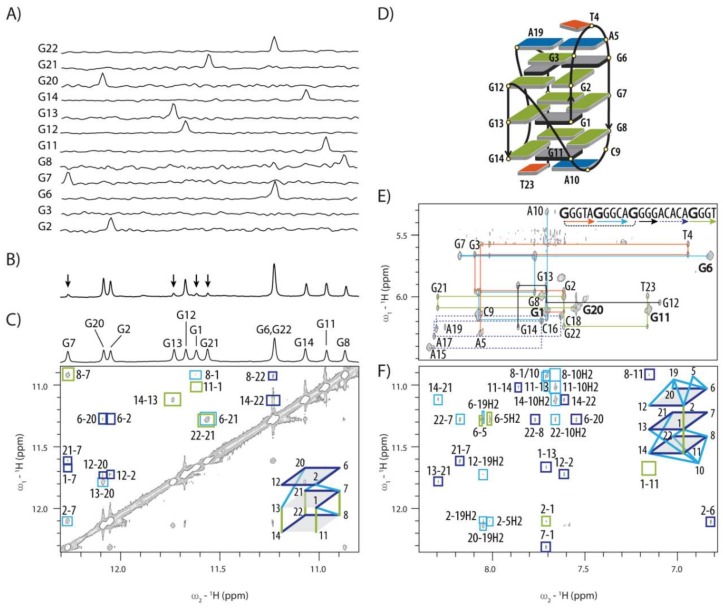
Topology determination of HPV52_(1–4)_. (**A**) 1D ^1^H-^15^N HSQC spectra of site-specific partially labelled oligonucleotides for identification of guanine residues in the G-quadruplex core. (**B**) Imino region of ^1^H-NMR spectrum 5 min after D_2_O-H_2_O exchange. Arrows point to the resonances of the least water accessible imino protons. (**C**) Imino-imino region of 2D NOESY spectrum (τ_m_ 250 ms) with assigned contacts between pairs of imino protons and schematics of correlations within G-quadruplex core. (**D**) Topology of HPV52_(1–4)_, where residues with extensive contacts to G-quadruplex core are shown with rectangles. Guanine residues in *syn* and *anti*-conformation, thymine and adenine residues are shown in dark grey, green, orange and blue colours, respectively. (**E**) Anomeric-aromatic region of 2D NOESY spectrum with sequential walk along the oligonucleotide sequence. (**F**) Imino-aromatic region of 2D NOESY spectrum with assigned connectivities between H1 and H8 pairs of protons within G-quadruplex core and contacts that define position of loop residues. Spectra were recorded at 25 °C, pH 7, 50 mM [K^+^] and c_oligo_ between 1.0 and 2.7 mM. 2D NOESY was recorded with τ_m_ of 250 ms.

Residue-specifically ^15^N isotope labelled oligonucleotides were used to resolve ambiguity as to which guanine residues of HPV52_(1–4)_ with uneven G-tract lengths (i.e., 3, 3, 4 and 3 nt) are involved in the G-quartet formation. Unexpectedly, only G1 and G2 from the first G-tract and all four residues from the third G-tract (i.e., G11, G12, G13 and G14) are incorporated in the G-quadruplex core (Figure 2A). We were able to establish topology model of HPV52_(1–4)_ through analysis of NOE contacts in the imino-imino region of 2D NOESY spectrum (Figure 2C,D). 

G1 is involved in the central G-quartet and is followed by G2 and G3-T4-A5 in edgewise loop orientation. G6-G7-G8 segment represents the second edge of the G-quadruplex core and adopts antiparallel orientation with respect to the first edge. Missing spot in the first edge of a G-quadruplex core defined by G1 and G2 is filled with G11, which is made possible by folding back of the DNA chain facilitated by C9 and A10 forming an edgewise loop. A no-residue V loop traverses the central G-quartet plane and connects G11 with G12-G13-G14 segment constituting the third edge. The loop consisting of A15-C16-A17-C18-A19 segment connects G12-G13-G14 and G20-G21-G22 edges of G-quadruplex core in a propeller-type orientation (Figure 2D).

Sequential connectivities in the anomeric-aromatic region of 2D NOESY spectra of HPV52_(1–4)_ are broken as expected at either *anti-syn* steps or in the loop regions (Figure 2E). Four instead of five strong intra-residual H1′-H8 cross-peaks denoting *syn* conformation are observed in NOESY spectrum for residues G1, G6, G11 and G20 (marked in bold in Figure 2E and colored grey in Figure 2D). G12 would be assumed to adopt *syn* conformation considering the established ‘rules’ on orientation of the strands within the G-quadruplex core [38], but it displays a weak H1′-H8 cross-peak and clearly adopts an *anti*-conformation. In full support, ^13^C-NMR chemical shifts of C8 atoms are downfield [39,40,41] for four guanine residues adopting predominantly *syn* conformation (Figure S3).

Several NOE contacts of imino protons of guanine residues involved in the G-quadruplex core with methyl group of T23 and H2 and H8 protons of A5, A19 and especially A10 suggest extensive nucleobase stacking of loop residues on G-quartets (Figure 2F). Altogether the observed NOE contacts indicate that the capping structures on the both sides of the G-quadruplex core formed by the two edgewise loops together with A19 and T23 add to the overall stability of the structure. Interestingly, capping structures do not increase protection of imino protons in the G-quadruplex core from exchange with bulk water as hydrogen exchange times for all residues in the two outer G-quartets are relatively short. Signals in the imino region of ^1^H-NMR spectrum are observed already 5 minutes after change of solvent from D_2_O to H_2_O (Figure 2B). Signals of guanine imino protons of the central G-quartet also re-appear relatively quickly, which shows that they are relatively poorly protected from exchange with bulk water molecules (Figure 2B). 

**Figure 3:**
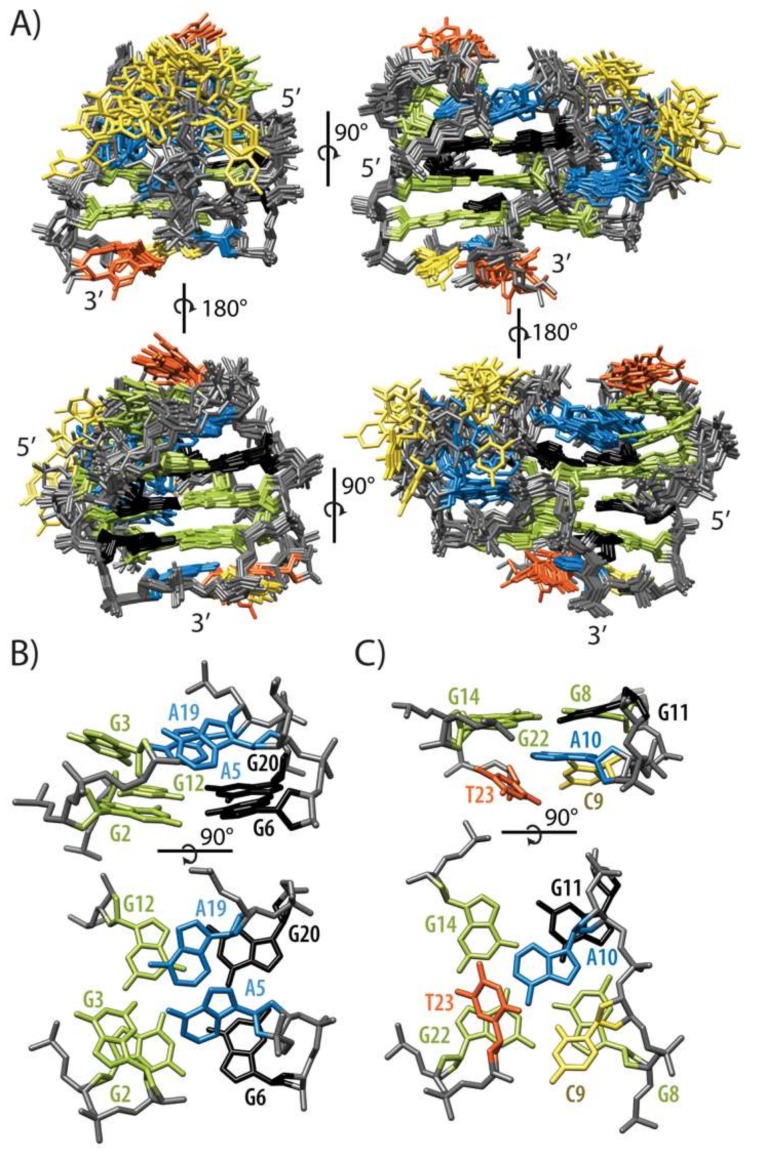
Structure of HPV52_(1–4)_. (**A**) Superposition of 10 lowest-energy structures of HPV52_(1–4)_. (**B**) Capping structure formed by G3, A5 and A19. (**C**) Capping structure formed by C9, A10 and T23. Guanine residues in *syn* and *anti*-conformation, thymine, adenine and cytosine residues are shown in black, green, orange, blue and yellow colour, respectively. DNA backbone is shown in grey.

High-resolution structure of HPV52_(1–4)_ (Figure 3A) was calculated with 377 NOE and 82 torsion angle restraints and is with the exception of C16–C18 region very well-defined (Table 1). Insertion of G1 in the middle of the G-quadruplex core introduces a loop extension, similar to the fold-back feature found in other G-quadruplexes. Hydrogen bond directionalities are in agreement with (3 + 1) topology and follow clockwise orientation for two G-quartets (G11→G14→G22→G8 and G1→G13→G21→G7) and anti-clockwise for one G-quartet (G2→G6→G20→G12). Stacking of G-quartets is based on 5/5-membered ring overlap between G1-G13-G21-G7 and G2-G6-G20-G12 quartets, and on 6/5-membered ring overlap between G1→G13→G21→G7 and G11→G14→G22→G8 quartets. Stacking of the loop residues onto the G-quadruplex core is especially pronounced for both capping structures. Capping structure formed by G3-T4-A5 edgewise loop is complemented by A19 from propeller loop at the opposite side of G2-G6-G20-G12 quartet (Figure 3B). On the other side of the G-quadruplex core, residues C9, A10 and T23 form capping structure that has been found to adopt two different conformations in the ensemble of calculated structures. In the first conformation that accounts for 70% of lowest energy structures, methyl group of T23 points away from G8-G11-G14-G22 quartet (Figure 3C). In the second, T23 lies in the plane with C9 and A10, while hydrogen bonds are formed between amino proton of A10 and O4 of T23 as well as between amino proton of C9 and O2 of T23. Even though cumulative length of loops for HPV52_(1–4)_ quadruplex is considerable, only three (T4, C16 and C18) out of 11 residues protrude from the structure. T4, however, is stacked with G3 and is quite well defined. C16 and C18, on the other hand, represent the least defined part of the 5-residue long propeller loop, while its three adenine residues are less flexible. A15 aligns in the plane of the central G-quartet, A17 is found in different positions under A15, while A19 is involved in the capping structure and interacts with G3 and A5 of edgewise loop (Figure 3B). It must be emphasized that several NOE contacts that could lead to a well-defined A15-C16-A17-C18-A19 loop conformation were not used in structure calculations due to spectral overlap. For example, interesting sequential-like H1′-H8 NOE contacts were observed between the A15 and A17 residues, suggesting that they are most likely stacked on each other and possibly aligned in the planes of G-quartets. 

Residues that display non-B-DNA ranges of sugar-backbone torsion angles cluster in the regions of sharp turns of DNA chain, most notably at A5, C9-A10 and G12-G13. Calculated structures showed that position of these residues was insufficiently defined by NOE contacts alone and that introducing torsion angles restraints importantly reduced their indeterminacy. For example, C9 is localized in the groove defined by residues G11-G1-G2 and G6-G7-G8 in several structures that were calculated without restraint for β and γ torsion angles of C9, although no NOE contacts between C9 and residues on the both sides of the groove were observed. Analysis of backbone torsion angles in those structures revealed that in-groove conformation shown to be most stable in MD simulations was not in agreement with experimentally determined β (*g^+^*) and γ (*t*) values of C9. Using β and γ torsion angle restraints led to C9 being positioned below G8-G11-G14-G22 quartet (Figure 3C). G12 is the only residue of HPV52_(1–4)_ that adopts the C3′-*endo* conformation, which may be attributed to the sharp turn of DNA chain and fits well with unusual torsion angles of G12 and G13.

### 2.3. Conformations of Edgewise G3-T4-A5 and A No-Residue V Loop Justify Unusual Chemical Shifts

**Figure 4:**
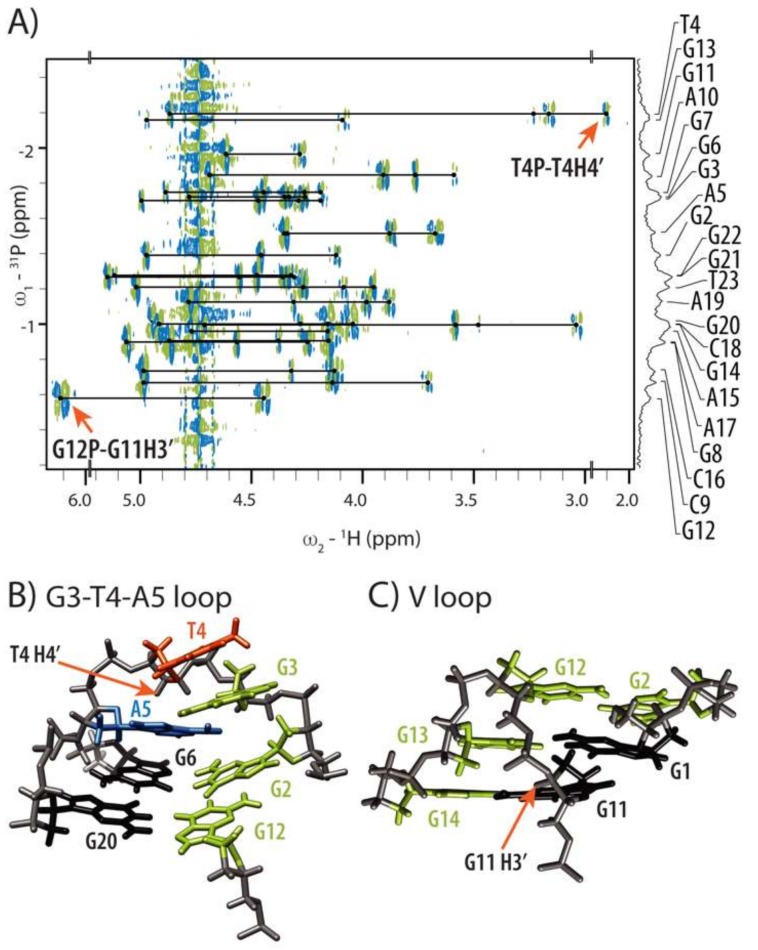
HP-COSY spectrum and structural details of G3-T4-A5 edgewise and no-residue V loop. (**A**) HP-COSY spectrum with assignment of phosphorus resonances in 1D ^31^P spectrum. (**B**) Close-up of G3-T4-A5 edgewise loop shows proximity of H4′ proton of T4 residue to aromatic ring of A5. (**C**) Perusal of V loop shows position of H3′ proton of G11 residue. Colour scheme used is the same as in Figure 3.

The observed NMR chemical shifts of HPV52_(1–4)_ exhibit several uncommon values which are however in perfect agreement with the calculated high-resolution structure. Most notable is the chemical shift of H4′ of T4 at δ 2.097 ppm (Figure 4A), which is shifted upfield by more than 2 ppm in comparison to the average chemical shift of H4′ of thymine residues (δ 4.14 ppm) [42]. As G3-T4-A5 edgewise loop conforms to the GNA loop sequence requirements [43,44,45,46,47,48], its distinct conformation brings T4 H4′ proton in close proximity of shielding zone of A5 (Figure 4B). Although resonance of G3 amino proton was not observed even at low temperatures, and therefore the sheared base pair between G3 and A5 that is typical for GNA loops could not be confirmed experimentally, conformation of G3 and A5 in calculated structures is very similar to a GA sheared base pair. Non-observed signal for amino group of G3 could be rationalized by the involvement of A19 in the dynamic hydrogen bonding network with G3 and A5 (Figure 3B). This could reduce the intensity of the signals corresponding to hydrogen-bonded protons of G3. Moreover, intermediate to fast exchange on NMR chemical shift time scale was observed from 0 to 40 °C for G3-T4-A5 residues formally constituting an edgewise loop (*vide infra*), which might have also precluded observation of signals corresponding to hydrogen bonded amino protons at low temperatures.

Second atypical chemical shift has been observed for G11 H3′, which is shifted downfield to δ 6.088 ppm into the region of anomeric protons (Figure 4A). Together with unusual conformations of torsion angles β (*g^+^*) and γ (*t*) of G13, α, ε and ζ torsion angles of G12 as well as C3′-*endo* puckering and *anti*-conformation of G12, atypical chemical shift of H3′ proton of G11 substantiates unusual conformation of G12-G13 part of DNA chain. Perusal of the calculated structures shows that sugar-phosphate backbone undergoes effectively a 180° turn at G12 in order to accommodate a no-residue V loop (Figure 4C). As a consequence, H3′ proton of G11 is positioned in close proximity of deshielding zone of its aromatic ring, which rationalizes its downfield shift. Furthermore, G11 H3′ proton is positioned also in the close proximity to H8 proton of G13 (3.5 Å), whose dipole-dipole interaction was indeed observed as a cross-peak in NOESY spectrum, albeit it was not used in structure calculations due to the spectral overlap.

### 2.4. Internal Motion of G3-T4-A5 Edgewise Loop

**Figure 5:**
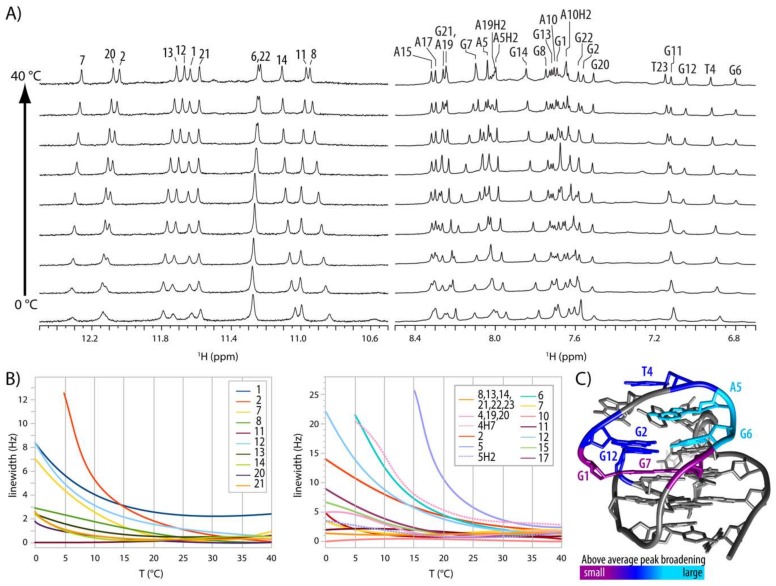
Spectral changes of HPV51_(1–4)_ as a function of temperature. (**A**) Imino and aromatic regions of 1D ^1^H spectra in 5 °C steps from 0 to 40 °C. (**B**) Temperature dependency of signal width at half-height of imino (left) and aromatic and methyl protons (right). To estimate contributions other than change of temperature and viscosity the minimal line broadening observed at certain temperature has been subtracted from measured values for specific residues. (**C**) Structure of HPV52_(1–4)_ with coloured residues displaying line-broadening above 10 and 15 Hz for imino and aromatic protons, respectively.

^1^H-NMR spectra recorded in 0 to 40 °C temperature range show that several imino and aromatic resonances of G1, G2, T4, A5, G6, G7 and G12 broaden severely and some even merge into the baseline at lower temperatures (Figure 5A). The largest changes in resonance linewidths are concentrated at A5-G6 and neighboring residues (Figure 5B,C). The largest signal broadening has been observed for A5, with its inter- and intra-residual NOESY cross-peaks involving sugar and aromatic protons practically non-observable below 15 °C. Signals for G6 also broaden dramatically, although effect on its H1 resonance could not be evaluated unambiguously due to the overlap with G22 H1. As *syn* to *anti* reorientations of G6 could be a possible cause for the observed dynamics, we prepared oligonucleotide with 8Br-dG residue at position 6. We reasoned that modification should prevent G6 from adopting *anti* conformation due to the sterically preferred orientation of large bromine atom away from the sugar moiety. Spectra of 8Br-G6-modified HPV52_(1–4)_ at 25 and 5 °C showed similar line broadening for imino resonances of the residues G1, G2, G20, G6, G7 and G12, while other residues were affected minimally (Figure S4B). However, we observed that imino resonances of G2-G6-G20-G12 quartet of G6-8Br HPV52_(1–4)_ were broadened at 5 and 10 mM potassium concentration (Figure S4A), which was attributed to slow formation of G2-G6-G20-G12 quartet as a result of incorporation of bulky G-8Br modification. In agreement with presumption of more open conformation of G2-G6-G20-G12 quartet, imino protons were more susceptible to exchange with water. Namely, cross-peaks of G20, G12 and G6 with water were observed for the G6-8Br HPV52_(1–4)_ oligonucleotide, but not for the parent HPV52_(1–4)_. To exclude slow G-quartet formation as the cause of spectral similarities with HPV52_(1–4)_ at low temperatures, 2D NOESY spectra of both oligonucleotides at 25 and 5 °C were compared. Analysis showed that 8Br-G6 modification did not prevent dynamics in the G3-T4-A5 loop, since H1′/H2′/H2″-H8 and H2′/H2″-H1′ cross-peaks of A5 of G6-8Br HPV52_(1–4)_ were not observed at 5 °C, while resonances of G12 and T4 were severely broadened and no additional cross-peaks appeared. As the same behavior was detected for HPV52_(1–4)_ (Figure 5), *syn-anti*-reorientation of G6 is not the cause of observed dynamics. In attempt to reach slow dynamic regime to be able to identify species in conformational exchange temperature was lowered to −10 °C or 1 M choline dihydrogenphosphate was added to the sample to increase viscosity. Nevertheless, similar spectral characteristic at those conditions indicated that exchange observed was still in intermediate regime. Interestingly, structural fluctuations presumably due to the wobbling of A residue between *syn* and *anti*-position was reported for GNA minihairpin loops [47], while for (GGA)_4_ G-quadruplex that consists of three consecutive GNA loops no reports of dynamic behavior exist [45,46]. 

### 2.5. GNA and Four Guanine Tract Are Crucial Structural Elements That Guide Folding of HPV52_(1–4)_

G3-T4-A5 segment with its predisposition to adopt a structure of a well-described GNA trinucleotide loop could drive conformation of HPV52_(1–4)_ into a preorganized state that is on the pathway for folding into a G-quadruplex, even more so since structuring of GNA loops is not [K^+^]-dependent in a way that is critical for G-quadruplex folding. Several modifications were introduced into HPV52_(1–4)_ in order to better understand sequence requirements for its structure (Figure S5). First, 4T > A modification in HPV52_(1–4)_ was used to examine effect of N residue in GNA loop on G-quadruplex stability. Yoshizawa et al. [47] have shown that the stacking capability of N residue onto the sheared GA base-pair correlates with GNA minihairpin stability with a 6 °C increase in Tm in the case of GAA compared to GTA loop. Thermal stability of HPV52(1–4) with 4T > A modification was therefore expected to increase. However, no substantial change in melting temperature of HPV52_(1–4)_ 4T > A compared to HPV52_(1–4)_ was observed, while NMR experiments clearly showed conservation of the fold (Figure S5). This suggests that contribution of GNA loop to the overall G-quadruplex stability is not as straightforward as in the hairpin-stem loop structures and depends on a wider structural context. Thymine residue in GTA loop in HPV52(1–4) could, for example, importantly facilitate initial stages of G-quadruplex folding if not even trigger it via molten globule-like state, as was shown for telomeric sequences [49]. 

Next, we examined the overall importance of 5A > T modification for conservation of HPV52_(1–4)_ fold, while keeping in mind that a mutated GTT loop does not conform to GNA sequence requirements. As expected, 5A > T modification was found to abolish formation of HPV52_(1–4)_ G-quadruplex and resulted in the formation of more than one structure (Figure S5B). These observations confirmed importance of GNA loop for structural integrity of G-quadruplex structure adopted by HPV52_(1–4)_.

Influence of interaction of GNA loop with residues in other loops for G-quadruplex formation was tested with deletion of A19 that is included in hydrogen bonding network of GTA loop. We hypothesized that if GTA loop on its own can stabilize or lead folding of HPV52_(1–4)_, deleting A19 should not affect HPV52_(1–4)_ structure. Surprisingly, A19 was found to play an important part in formation of capping structure together with GNA loop, since deletion of A19 did not retain HPV52_(1–4)_ G-quadruplex fold (Figure S5B).

Finally, interruption of four guanine tract with T12 insertion was designed to test if G4 tract is actually beneficial for G-quadruplex formation, or does it complement formation of G-quadruplex framework that is set up by other very stable or faster forming structural elements. Titration of the oligonucleotide with T12 insertion with aqueous KCl prevented formation of HPV52_(1–4)_, G-quadruplex and resulted in several different structures (Figure S5), which leads us to conclude that G4 tract and resulting V loop are important for formation of HPV52_(1–4)_ G-quadruplex.

## 3. Discussion

G-rich sequence from genome of HPV type 52 which is the cause of 20% of cervical cancer comprises five G-rich tracts. Their number and variation in length (3, 3, 4, 3 and 3 nt) lead to folding into several different G-quadruplex structures. We could detect at least five different monomeric folds for HPV52_(1–5)_ with the help of analysis of imino fingerprint region and naturally occurring SNPs [10]. In order to unveil and possibly determine structure(s) of predominant forms involved in dynamic equilibrium we focused on oligonucleotide HPV52_(1–4)_ that consists of the first four G-tracts and which demonstrated promising preliminary NMR data. High-resolution NMR structure of a G-quadruplex adopted by 23 nt HPV52_(1–4)_ has several exciting structural features, in line with observation of unusual NMR parameters. G1 is found in the central G-quartet stacked between G2 and G11, which constitute one of the outer G-quartets each. Formally, G1–G11 segment adopts a circular arrangement with G11-G1-G2 and G6-G7-G8 forming edges of a G-quadruplex adopted by HPV52_(1–4)_. A double fold-back of DNA chain is made possible by three (G3-T4-A5) and two (C9-A10) residue edgewise loops that span the opposite sites of the same wide groove. At the same time, G11 is in the terms of primary sequence part of the four consecutive guanine residues (i.e., G11–G14) that are all included in the three G-quartet core and are connected via a no-residue V loop. The last loop consisting of A15-C16-A17-C18-A19 connects G12-G13-G14 and G20-G21-G22 edges of G-quadruplex core in a propeller type orientation. Perusal of G-quadruplex adopted by HPV52_(1–4)_ suggests that capping structures on both sides of the three G-quartet core contribute substantially to its stabilization. In particular, G3-T4-A5 edgewise loop in the GNA loop conformation interacts with A19 at the 5′-end, while C9-A10 edgewise loop interacts with T23 at the 3′-end.

The structure of HPV52_(1–4)_ is similar to a G-quadruplex adopted by 5′ intronic sequence of gene *chl1*, whose product belongs to a FANCJ helicases. The *chl1* G-quadruplex has been reported earlier when searching for sequence pattern G_3_-T-G_4_-AA-G_4_-T-G_3_T [50]. However, HPV52_(1–4)_ does not adhere to G_3_-N-G_4_-NA-G_4_-A/C/T-G_3_-A/C/T sequence requirements that were at that time recognized as vital for adopting the fold. In fact, HPV52_(1–4)_ has only the following few characteristics in common: (i) the 5′-end G_3_ tract of which only the first two guanine residues are involved in the G-quadruplex core, (ii) an NAG_4_ tract that incorporates a no-residue V-loop, and (iii) a thymine residue at the 3′-end. Updated sequence requirement for folding of HPV52_(1–4)_ and *chl1* into their respective G-quadruplex structures is G_3_-N_n_-G_3_-N_n_A-G_4_-N_n_-G_3_-A/C/T, where n ≥ 1 and N can be any nucleotide. Furthermore, HPV52_(1–4)_ and *chl1* allow us to better understand importance of specific structural elements that stabilize the fold. From comparison of the two structures it can be concluded that the length of both edgewise loops spanning the wide groove may vary and any structural elements formed within the two edgewise loops contribute to additional stabilization of the structure, but are not essential for folding. However, considering that the two-residue edgewise loops spanning wide grooves were shown to be very rare due to imposing the strain in structure [51], it is highly unlikely that two short (2 nt) loops could be accommodated in the same structure. As a consequence, the minimal cumulative length of both edgewise loops of a double fold-back circular element is presumably five residues. Interestingly, the sugar-phosphate backbone conformation of a two-residue edgewise loop bridging wide groove is the same for both HPV52_(1–4)_ (C9-A10 loop) and *chl1* (G3-T4 loop). Very similar sugar-phosphate backbone conformation was observed in the structure of d(G_4_C_2_)_3_G_4_), the only other known experimentally observed example of a two-residue edgewise loop (C11-C12) spanning a wide groove (PDB ID 2N2D) (Figure S6) [52]. In contrast to the experimentally determined structures with both residues of the loop positioned above a G-quartet, MD simulations have shown the first residue of a two-residue edgewise loop to reside in the wide groove [51]. It must be emphasized that in-groove conformation was observed for C9 during structure calculations of HPV52_(1–4)_ when experimentally determined restrains for backbone torsion angles of C9 were excluded from structural calculations, which shows that other interactions within the loop must compensate for energetically less favorable loop conformation. 

For HPV52_(1–4)_ the first edgewise loop was recognized as a very stable GNA type of loop. Modification of oligonucleotide sequence, however, showed that this loop stabilized fold together with A19 residue from the propeller loop stacking on G2-G6-G20-G12 quartet. Interestingly, the second edgewise loop of *chl1* with sequence G8-A9-A10 can also fold as GNA type loop. Indeed, sheared GA base-pair was detected for *chl1* and although chemical shift of A9 H4′ proton was not reported, the conformation of the loop fits to the GNA type loop. Sheared GA base-pair is involved in triple with 3′-end residue (T19 and A19), which means that in both *chl1* and HPV52_(1–4)_ structures additional interactions with other residues involve GNA type of loop in the capping structures.

The most defining part of HPV52_(1–4)_ and *chl1* structures seems to be AG_4_ tract that results in A10 residue being stacked on the outer G-quartet and thus contributing to the stabilization of a no-residue V loop. To the best of our knowledge, HPV52_(1-4)_ represents the third structure with such a loop, the first one being found in the dimeric d(G_3_T_4_G_4_)_2_ [53]. In comparing structures of V loops it becomes clear, however, that they differ in conformation of their sugar-phosphate backbone (Figure S7). Namely, HPV52_(1–4)_ is the only case in which H3′ of G11 is observed at δ 6.088 ppm and in which this proton is placed in the plane and close to the aromatic ring of G11, resulting in considerable deshielding related to the decreased electron density. While structures of HPV52_(1–4)_ fall into two different groups with regards to the sugar-phosphate backbone torsion angle values of G11-G12, spatial position of sugar-phosphate backbone changes minimally within the two groups and position of H3′ of G11 is conserved (Figure S7). Several conformations of sugar-phosphate backbone between G11 and G12 were found also for *chl1*, but while H3′ of G11 is placed close to the aromatic ring in *chl1*, it is not in plane with it, as in HPV52_(1–4)_. Therefore, δ (H3′) of G11 in *chl1* is somewhat, but not substantially downfield shifted (δ 5.62 ppm) [50]. For d(G_3_T_4_G_4_)_2_, H3′ of G19 is placed far away from its aromatic ring and its chemical shift (δ 5.00 ppm) is close to the average value of H3′ chemical shifts of guanine residues (δ 4.95 ppm) [42]. 

Another interesting feature of V loop region of G-quadruplex adopted by HPV52_(1–4)_ is that sugar ring of G12 predominantly adopts North-type conformation, while it is 80% South in d(G_3_T_4_G_4_)_2_ [53] and no data has been reported for *chl1* [50]. The difference is also in glycosidic torsion angle χ of G12, which is in *anti*-region for HPV52_(1–4)_ and *chl1*, but in *high*-*anti* region for G20 of d(G_3_T_4_G_4_)_2_. As North conformation of sugar ring is energetically less favorable for DNA, structural flexibility must be additionally restricted for HPV52_(1–4)_ in comparison to d(G_3_T_4_G_4_)_2_. In summary, while HPV52_(1–4)_ shares a no-residue V loop with the two other known G-quadruplexes, structural details show existence of several distinct loop subtypes, which is rather unanticipated for such a short structural element. We hypothesize that combination of a two-residue edgewise loop that traverses wide groove and a no-residue V loop in HPV52_(1–4)_ represents two consecutive strained elements that impose spatial limitations on the structure and result in the accumulation of unusual and, in a classical view, energetically less favorable torsion angles conformations. 

Our study has been focused on formation of G-quadruplex structures formed by G-rich sequence of HPV type 52. Recently, increasingly large efforts have been made by G-quadruplex community to understand biological role of G-quadruplexes, while structural part of this effort is complicated by polymorphism and challenges in relating a specific (element of) structure to a specific function. If, however, G-quadruplexes are ever to be used as targets for rational drug design, structural information is of paramount importance. This knowledge is also vital for our understanding of processes that G-rich sequences found in various parts of genomes are involved in. Moreover, while number of detailed 3D structures of G-quadruplexes has been increasing steadily, we are still very far from understanding their folding or polymorphism of even quite simple G-rich sequences, such as HPV52_(1–4)_. SNPs were proven useful to better understand structural polymorphism of HPV52_(1–4)_, albeit G-quadruplexes with SNPs are supposedly less relevant in biological context as they form structures with lower melting temperatures and were, as expected for SNPs, found in only a very low number of isolates. While G-quadruplex structures formed by G-rich HPV52 oligonucleotides display high enough thermal stability to reduce expression levels in usual in vitro transcription experiments, the high complexity of the HPV viral life cycle precludes us from drawing any (definite) conclusions from simplified model systems. More comprehensive understanding of impact of G-quadruplex formation on gene expression of HPV52 will therefore require further and more complex studies. Nevertheless, it is tempting to speculate that formation of a certain structure would depend on the direction in which the DNA chain is unwound by DNA processing enzymes, as it would expose single stranded DNA at either 5′ or 3′ ends of G-rich sequence, corresponding to HPV52_(1–4)_ and HPV52_(2–5)_ oligonucleotides, respectively. Formation of a HPV52_(1–4)_ structure with G1 in the central G-quartet opens a question of relevance of such structure within DNA of viral genome. However, increasing the length of HPV52_(1–4)_ at both 5′ or 3′ ends does not preclude G-quadruplex formation (Figure S5), suggesting that the G-quadruplex structure described herein can form even in the context of longer DNA chain. Moreover, HPV52_(1–4)_ offers several structurally distinct elements besides G-quartet planes that are common to all G-quadruplexes and are typically targeted with ligands. For example, while narrow groove that accommodates a no residue V-loop is inaccessible for HPV52_(1–4)_, both medium and wide grooves are completely accessible for hydrogen bond recognition of the G-quartet edges (Figure S8). Moreover, capping structures on both sides efficiently lengthen wide and medium grooves between G20-G22 and G6-G8 tracts, while A15-C16-A17-C18-A19 loop defines a pocket in medium groove between G12-G14 and G20-G22 tracts (Figure S8) that is particularly interesting potential target.

## 4. Materials and Methods 

### 4.1. Sample Preparation 

Oligonucleotides were either purchased from Eurogentec (Seraing, Belgium) and Metabion (Planegg, Germany) or synthesized on a DNA/RNA Synthesizer H-8 (K&A Laborgeraete GbR, Schaafheim, Germany) using standard phosphoramidite solid-phase chemistry. Cleavage of protecting groups was carried out in 1:1 solution of methylamine and aqueous ammonia at 65 °C for 20 min. All samples were purified and desalted with the use of a Millipore Stirred Ultrafiltration Cell model 8010 (Cole-Parmer, Vernon Hills, Illinois, USA). Samples were prepared by dissolution in H_2_O containing 10% of ^2^H_2_O. pH was adjusted to 6.8 or 7.0 with LiOH solution and held constant with 10 mM potassium phosphate buffer (pH 6.8/7.0). Aqueous KCl was titrated into the samples to the final concentration of 50 mM. For measurements at −10 °C oligonucleotide sample was dissolved in 20% deuterated aqueous methanol, while for measurements in choline dihydrogen phosphate lyophilized sample was dissolved in its 1M solution. Annealing procedures included heating of the sample to 95 °C for 3 min and slow cooling to room temperature overnight. Strand concentration in the samples was ranging from 0.26–2.7 mM and was determined by UV absorption at 260 nm using CARY-100 BIO UV-VIS spectrophotometer (Varian, Santa Clara, CA, USA) and the computer program UV WinLab. Extinction coefficients used were 2.80 × 10^5^, 2.36 × 10^5^ and 2.28 × 10^5^ l mol^−1^ cm^−1^ for HPV52_(1–5)_, HPV52_(2–5)_ and HPV52_(1–4)_, respectively, and were determined by the nearest neighbour method.

### 4.2. CD Spectroscopy

The CD spectra are the average of five scans and were recorded on an Chirascan CD spectrometer (Applied Photophysics, Leatherhead, Surrey, UK) at 25 °C using a 0.1 cm path length quartz cell. The wavelength was varied from 200 to 320 nm in 1 nm steps. Samples for CD measurements were prepared at 10 μM oligonucleotide concentration in 10 mM potassium phosphate buffer and 40 mM KCl. A blank containing only 10 mM potassium phosphate buffer and 40 mM KCl was used for baseline correction.

### 4.3. UV Spectroscopy

UV melting curves were recorded with a Varian CARY-100 BIO UV/VIS spectrophotometer equipped with Cary Win UV Thermal program in cuvettes with 1 and 0.5 cm path-length at 260, 295 and 350 nm. Temperature interval was 70 °C or 80 °C with 0.1 °C/min temperature change. Measurements started at 10 °C for thermally susceptible samples (HPV52_(1–4)_ G22 > A and HPV52_(2–5)_ G22 > A) and at 90 °C for all other samples and were repeated four times. Samples were covered with mineral oil and stopped to prevent evaporation at high temperatures. Stream of nitrogen was applied throughout the measurements to prevent condensation at low temperatures. Sample concentration was 10 μM in 10 mM KPi/40 mM KCl.

### 4.4. Native Gel Electrophoresis

Polyacrylamide gel electrophoresis was carried out in temperature-controlled vertical Protean II XI Cell with PowerPac 3000 power supply machine (BioRad, Hercules, CA, USA) at 10 °C for 22 h at 120 V. Gel concentration was 15% (19:1 monomer to bis ratio). Gel was run in 25 mM Britton-Robinson buffer, pH 7, and 50 mM KCl. Sample concentration was 0.24 mM. DNA was visualized with Stains-all (Sigma Aldrich, St. Louis, MO, USA) staining. GeneRuler Ultra Low Range DNA ladder with 12-300 base pairs (Thermo Scientific, Waltham, MA, USA) was used as a relative mobility marker. Gel was photographed with a D3200 camera (Nikon, Minato, Tokyo, Japan).

### 4.5. NMR Spectroscopy

NMR spectra were recorded on VNMRS 600 and 800 MHz NMR spectrometers (Agilent-Varian, Santa Clara, CA, USA) in the temperature range 0–45 °C. DPFGSE pulse sequence was used to suppress the water signal. 2D NOESY spectra acquired at τ_m_ of 80, 150 and 250 ms were used to determine glycosidic torsion angle conformation, to establish oligonucleotide topology and consequently to assign exchangeable and non-exchangeable proton resonances. 2D DQF-COSY and TOCSY (τ_m_ of 20 and 80 ms) spectra were used to cross-check assignment of 2D NOESY spectra and to estimate sugar conformations.

Spectra were processed with programs VNMRJ (Agilent Technologies, Santa Clara, CA, USA) and NMRPipe [54]. Cross-peak assignment and integration with Gaussian fit procedure was achieved using software NMRFAM-SPARKY (NMRFAM) [55,56]. NOE distance restraints for non-exchangeable protons were obtained from 2D NOESY spectra (τ_m_ 80, 150 and 250 ms) recorded at 25 °C in 100% ^2^H_2_O and 10% ^2^H_2_O/90% H_2_O. Non-overlapping peaks only were used for the distance restraints calculations. Average volume of H7-H6 cross-peaks of T4 and T23 was used as reference distance of 3.0 Å [57,58]. Cross-peaks were classified as strong (1.8–3.6 Å), medium (2.5–5.0 Å) and weak (3.6–6.5 Å). Another 0.5 Å was added for restraints for ambiguous geminal protons (H2′/H2″ or H5′/H5″), as restraint was placed on a C atom (either C2′ or C5′). NOE distance restraints for exchangeable protons were obtained from 2D NOESY spectra recorded at 25 °C in 10% ^2^H_2_O/90% H_2_O with mixing times of 80, 150 and 250 ms. Cross-peaks of medium and weak intensity that could be observed in 2D NOESY spectrum with a mixing time of 80 ms were classified as strong (1.8–4.1 Å) and medium (2.5–5.5 Å), respectively. Cross-peaks that appeared in 2D NOESY spectrum with a mixing time of 150 and 250 ms were classified as weak (3.6–7.0 Å).

Data at 25 °C only were used in structure calculations, even though spectra recorded at 0 °C displayed several additional cross-peaks with exchangeable protons. However, as part of the structure was shown to be dynamic, the intensity of NOE cross-peaks at lower temperatures was considered unreliable. Torsion angle χ was restrained to *syn* (0 ± 90) for G1, G6, G11 and G20 and to *anti* region (240 ± 70) for all other residues, except A5 and A15. High intensity of the H1′–H8 cross-peak for A15 showed this residue might be involved in *syn-anti* equilibrium and was therefore left unrestrained, while residue A5 was left unrestrained due to the dynamic nature of the T4-A5-G6 region. Backbone torsion angles were restrained to typical values of g^+^/g^−^, t, g^+^, t and g^+^/g^−^ for α (G2, G3, G6, G7, G21), β (G2, G3, T4, G11, G12, G14, G21, G22), γ (G2, G3, T4, G8, A10, G14, A19, G21), ε (G1, G7, G8, G9, A10, G11, G14, A15, C16, A17, C18, A19, G21, G22) and ζ (G2, G3, G6, G7, G21) based on phosphorous chemical shift (α and ζ), visible 31P-H4′ cross-peak (β and γ) and strong 31P-H3′ cross-peak (ε) [46,59,60,61,62,63]. For A5, C9, A10 and G13 strong P-H5′/H5″ cross-peaks were observed, which indicate unusual rotamers of β. Unusual splitting patterns (−−++) of these residues were then compared with simulated HP-COSY cross-peaks in the Pikkemaat and Altona paper [62], which lead to limiting β to g^+^ for A5, C9, G13 and g^+^/t for A10. Intense H4′-H5′ or H4′-H5″ cross-peaks in DQF-COSY spectrum for residues A5, C9, G13 and G20 indicated that their γ torsion angle values are not in typical g^+^ range. Strong intensity of H2′/H2″-H5′/H5″ NOE cross-peaks and similar intensity of H3′-H5′ and H3′-H5″ cross-peaks in combination with intense H5″-H8 cross peaks for residues in *anti*-conformation indicated t conformation of γ torsion angle, which was determined for C9, G13 and G20. For A5, g^−^ conformation could not be excluded, which is why γ torsion angle of A5 was restrained to g^−^/t to exclude typical g^+^ values. Torsion angles that were shown to be involved in equilibria between several distinct conformations on the basis of spectral characteristics were left unrestrained.

### 4.6. Structure Calculations

Structure calculations were performed with CUDA version of pmemd module of AMBER 14 software [64], and parmbsc0 [65] force field with χ_OL4_ [66] and εζ_OL1_ [67] corrections. The initial extended single-stranded DNA structure was obtained with tleap program of AMBER14. Pairwise generalized Born implicit model was used with 0.4 fs time steps and collision frequency of 5 ps^−1^. For each simulated annealing (SA) a random velocity was used. The cut-off for non-bonded interactions was 999 Å and the SHAKE algorithm for hydrogen atoms was used. Solution-state structure was calculated in two steps of NMR restrained SA simulations. Topology was built in the first step with the help of restraints for hydrogen bonds, G-quartet planarities and limited number of H1-H1 and H6/8-H6/8 distance restraints. 1000 final structures were then calculated in 300 ps SA with the following temperature program: temperature was raised from 300 to 1000 K in the first 5 ps, held constant at 1000 K for 65 ps and scaled down to 0 K in the next 235 ps. Restraints used in the calculation were hydrogen bond (force constant 30 kcal mol^−1^ Å^−2^, 0–300 ps) and NOE-derived distance restraints (force constant 10 kcal mol^−1^ Å^−2^, 100–300 ps ), torsion angles χ, υ_2_, α, β, γ, ε and ζ restraints (force constant 200 kcal mol^−1^ rad^−2^, 0–300 ps) and planarity restraints for G-quartets (force constant 20 kcal mol^−1^ rad^−2^, 50–290 ps). All restraints were linearly increased to their final value in the first 100 ps of SA. A family of ten structures was selected based on the lowest energy and the smallest restraints violations. Structures were minimized with 100,000 cycles of steepest descent minimization. No planarity restraints were used in the final stage of structure refinement.

Atomic coordinates and chemical shifts for the reported NMR structure have been deposited with the Protein Data bank under accession number 5O4D and with the Biological Magnetic Resonance Bank under accession number 34145.

## Figures and Tables

**Table 1:** Structural statistics.

NMR Distance and Torsion Angle Restraints			
NOE-derived distance restraints	Non-exch.	Exch.	All
Total	322	55	377
Intra-residue	215	0	215
Inter-residue	107	55	162
Sequential	88	15	103
Long-range	19	40	59
Chemical shift derived distance restraints	4		
Hydrogen bond restraints	24		
Hydrogen bonds non-observed	3		
Torsion angle restraints	82		
G-quartet planarity restraints	36		
Structure statistics			
Violations			
Mean NOE restraint violation (Å)	0.14 ± 0.001		
Max. NOE restraint violation (Å)	0.33		
Max torsion angle restraint violation (°)	6.768		
Deviation from idealized geometry			
Bonds (Å)	0.012 ± 0.000		
Angles (°)	2.43 ± 0.03		
Pairwise heavy atom RMSD (Å)			
Overall	1.779		
G1-G14 + G20-G23	0.987		
G-quartets	0.751		

## Supplementary Materials

The following are available online at https://www.mdpi.com/1420-3049/24/7/1294/s1, Figure S1: HPV52 oligonucleotides resolved by PAGE electrophoresis, Figure S2: Melting profiles [68] of HPV52 oligonucleotides at 295 nm, Figure S3: C8 chemical shift differences for guanine residues, Figure S4: NMR characterization of G6-8Br modified HPV52_(1–4)_, Figure S5: Extended and modified sequences of HPV52_(1–4)_ and their NMR characterization, Figure S6: Comparison of 2-residue edgewise loops spanning the wide groove in HPV52_(1–4)_, *chl1* and (G_4_C_2_)_3_G_4_ G-quadruplexes, Figure S7: Comparison of a no-residue V loops in HPV52_(1–4)_, *chl1* and (G_3_T_3_G_4_)_2_ G-quadruplexes, Figure S8: Surface representation of grooves of HPV52_(1–4)_.
