# Supplementary material for: Towards Understanding of Polymorphism of the G-rich Region of Human Papillomavirus Type 52
Source: Molecules. 2019 Apr 2;24(7):1294. doi: 10.3390/molecules24071294 (PMC6479982; doi:10.3390/molecules24071294)
Supplement: Supplementary file 1 [file molecules-24-01294-s001.pdf]

SUPPLEMENTARY MATERIAL

Towards understanding of polymorphism of G-rich region of human papillomavirus type 52 by NMR

Maja Marušič [1] and Janez Plavec [1,2,3,*]

[1] Slovenian NMR Center, National Institute of Chemistry, Hajdrihova 19, SI-1000 Ljubljana, Slovenia

[2] EN-FIST Center of Excellence, SI-1000 Ljubljana, Slovenia

[3] Faculty of Chemistry and Chemical Technology, University of Ljubljana, SI-1000 Ljubljana, Slovenia

**To whom correspondence should be addressed: janez.plavec@ki.si.*

Table of contents

Figure S1: HPV52 oligonucleotides resolved by PAGE electrophoresis 3
Figure S2: C8 chemical shift differences 4
Figure S3: Characterization of G6-8Br modified $HPV52_{(1-4)}$ 5
Figure S4: Modified sequences of $HPV52_{(1-4)}$ and their NMR characterization. 6
Figure S5: Comparison of a 2-residue edgewise loops in $HPV52_{(1-4)}$, *chl1* and $(G_4C_2)_3G_4$................ 7
Figure S6: Comparison of a no-residue V loops in $HPV52_{(1-4)}$, *chl1* and $(G_3T_3G_4)_2$ 8
Figure S7: Surface representation of grooves of $HPV52_{(1-4)}$ 9

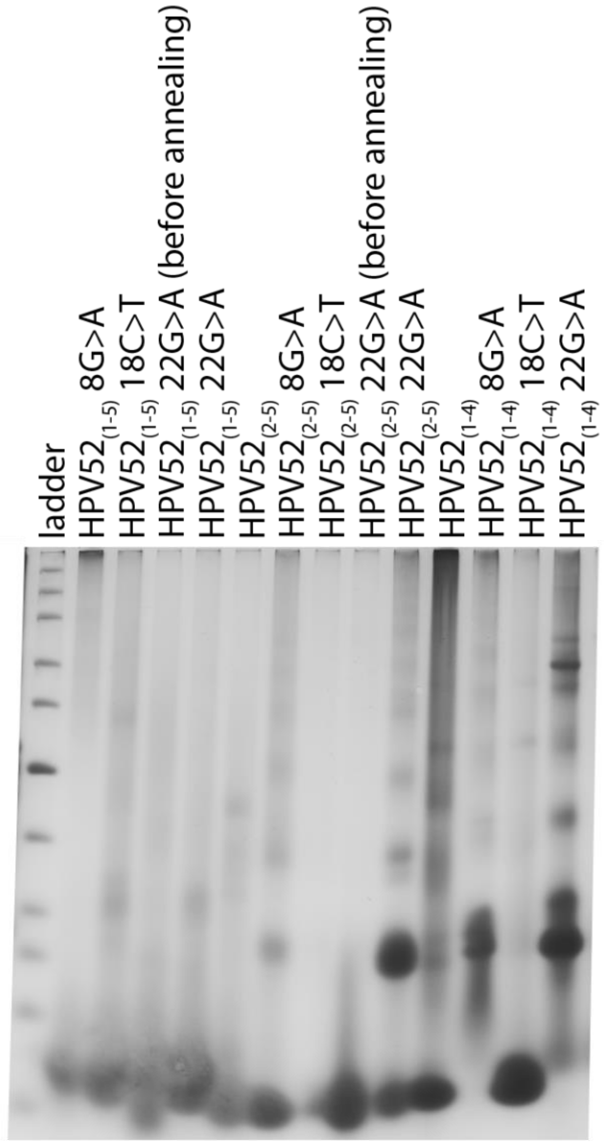

Figure S1: HPV52 oligonucleotides resolved by PAGE electrophoresis. Electrophoretic separation was performed in a 15% polyacrylamide gel at 10 °C in 25 mM Robinson buffer (pH 7.0) and 50 mM KCl. GeneRuler DNA ladder with 12-300 base was used as a relative mobility marker. Samples were, unless otherwise specified, loaded on a gel after annealing procedure.

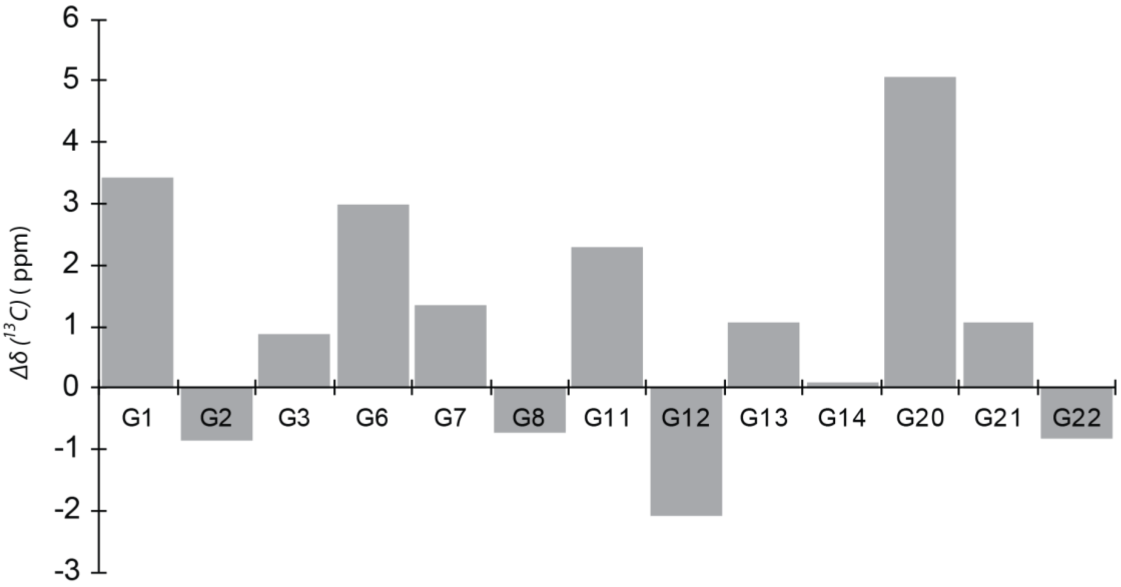

Figure S2: C8 chemical shift differences ($\Delta\delta(^{13}C) = \delta$ (^{13}C8 of a residue) - δ (average ^{13}C8 of residues in *anti* conformation) for guanine residues.

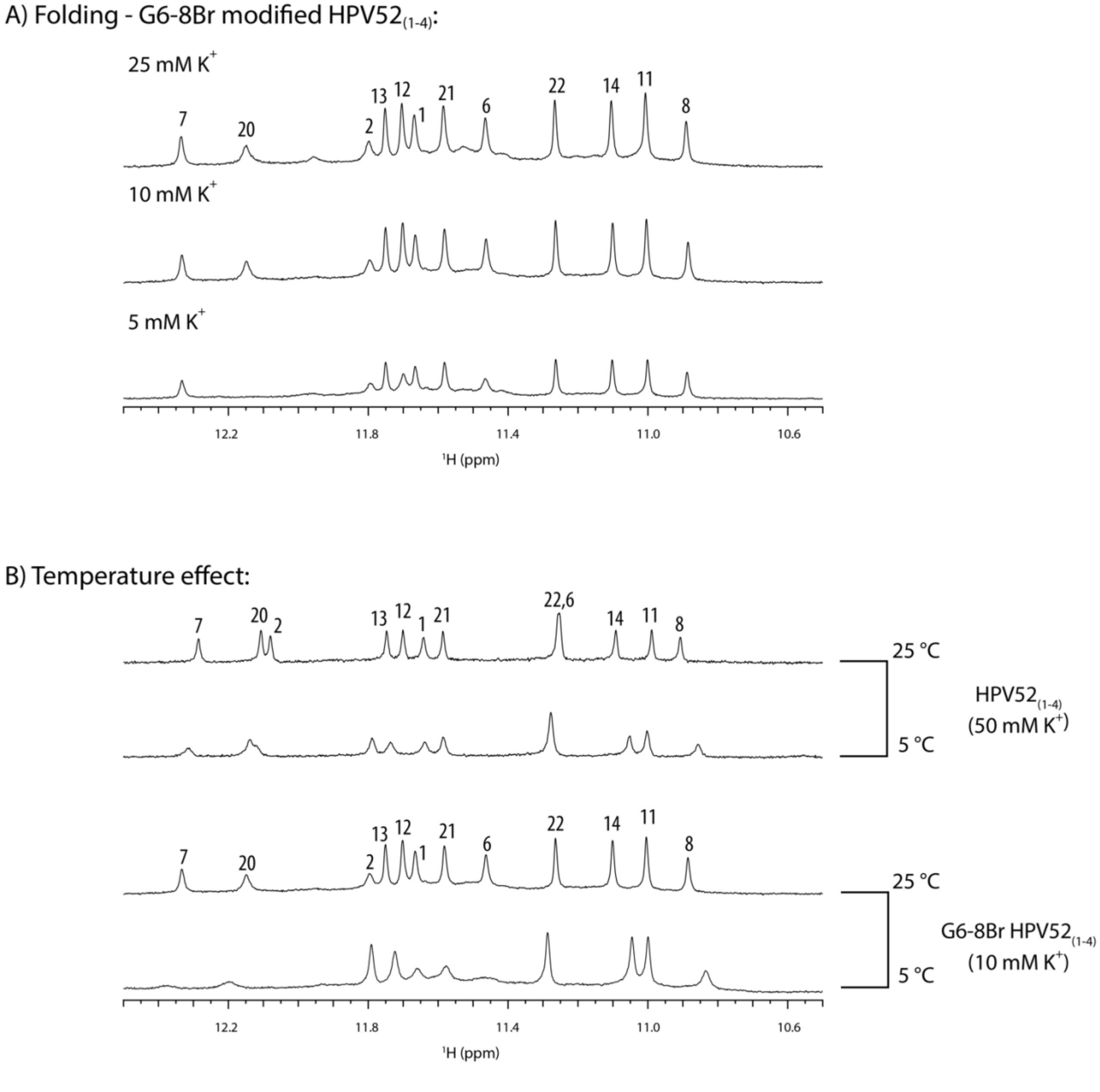

Figure S3: NMR characterization of G6-8Br modified HPV52(1-4). A) Slow folding observed for modified oligonucleotide, especially in the G2-G6-G20-G12 quartet. B) Temperature behaviour of G6-8Br HPV52(1-4) in comparison to HPV52(1-4).

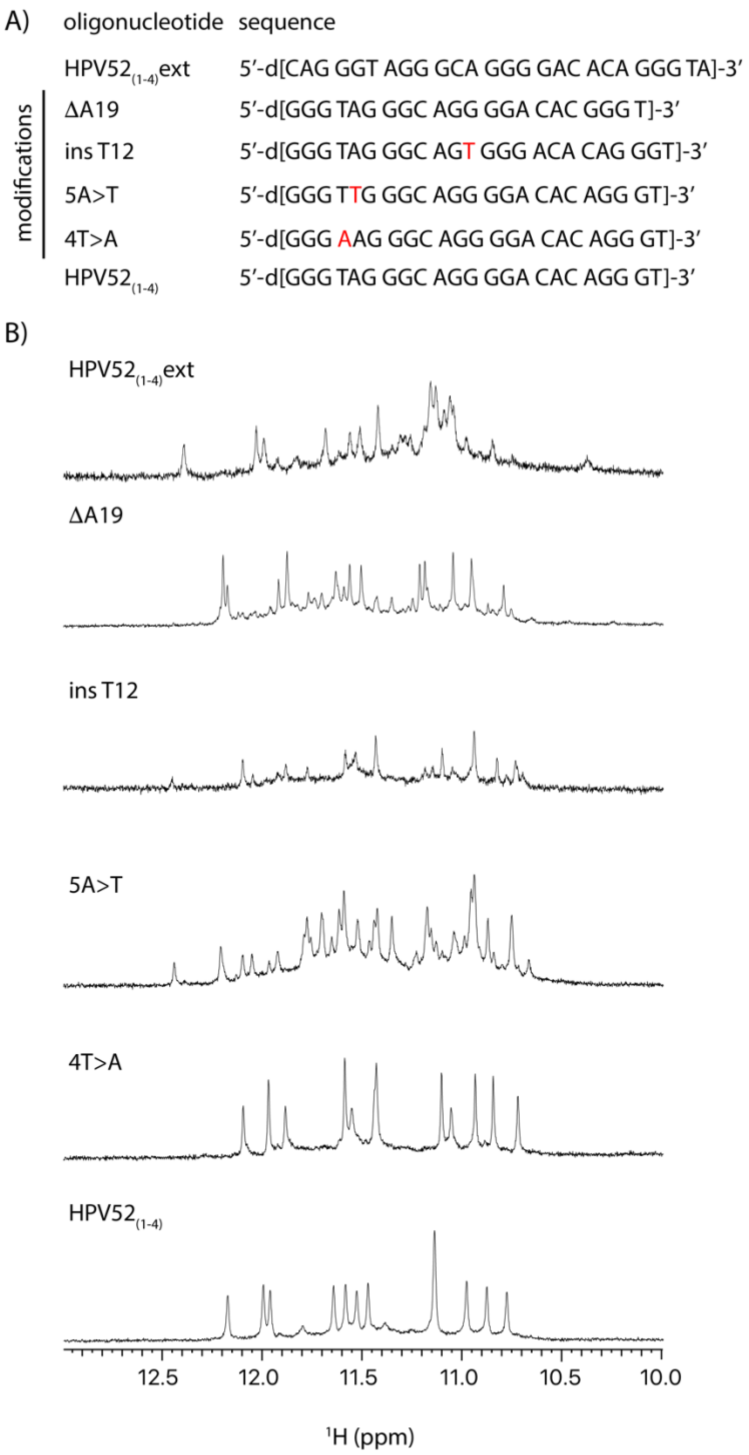

Figure S4: Extended and modified sequences of $HPV52_{(1-4)}$ and their NMR characterization. A) Sequences of $HPV52_{(1-4)}$ with extension or modifications. B) Imino regions of 1D ^{1}H NMR spectra of modified oligonucleotides.

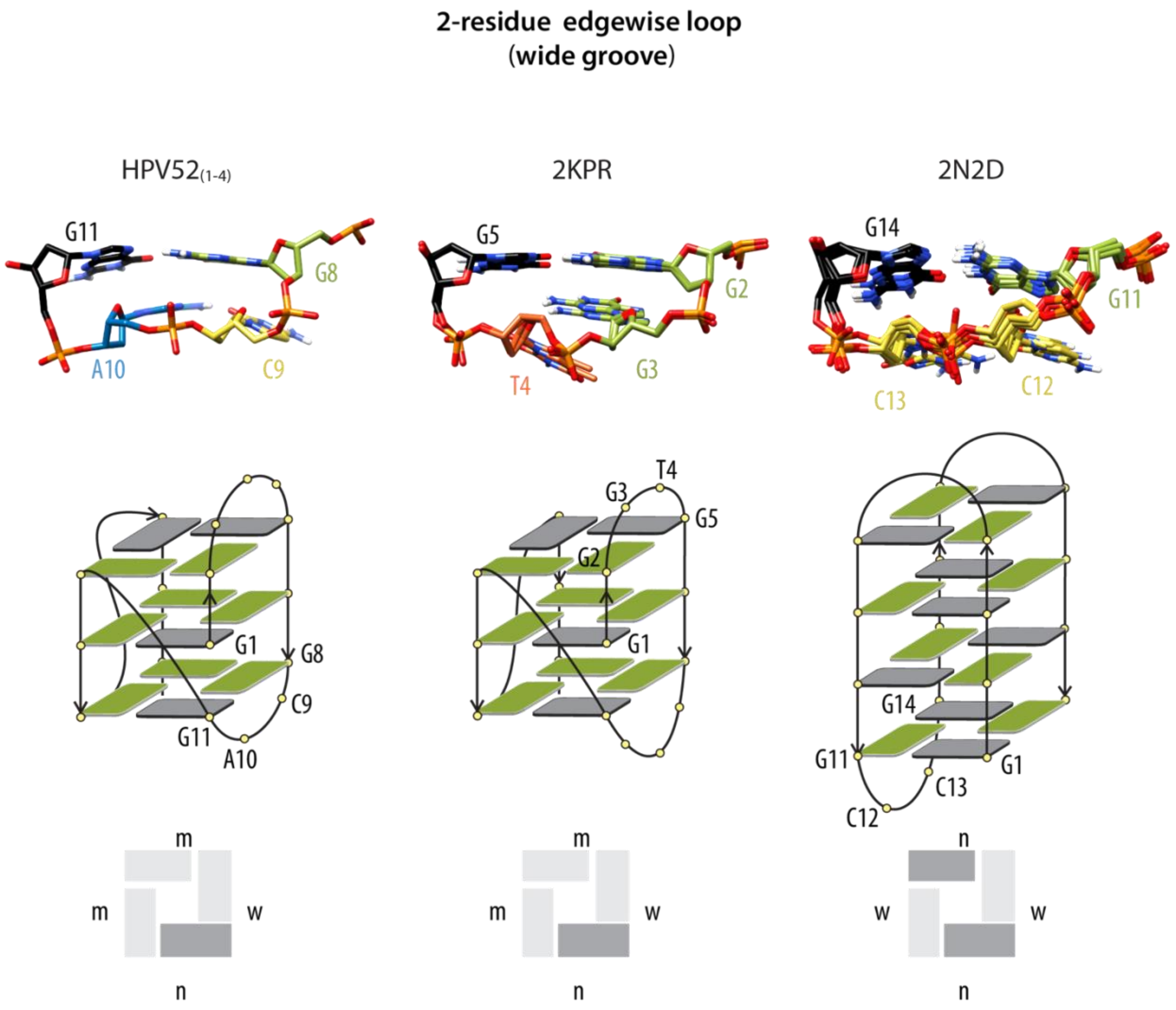

Figure S5: Comparison of 2-residue edgewise loops spanning the wide groove in HPV52$_{(1-4)}$, *chl1* (pdb code 2KPR) and $(G_4C_2)_3G_4$ (pdb code 2D2N) G-quadruplexes. Structural details of the loop, topology and distribution of groove widths are shown for each structure. All different conformations with regard to the backbone angles observed in the structures are displayed.

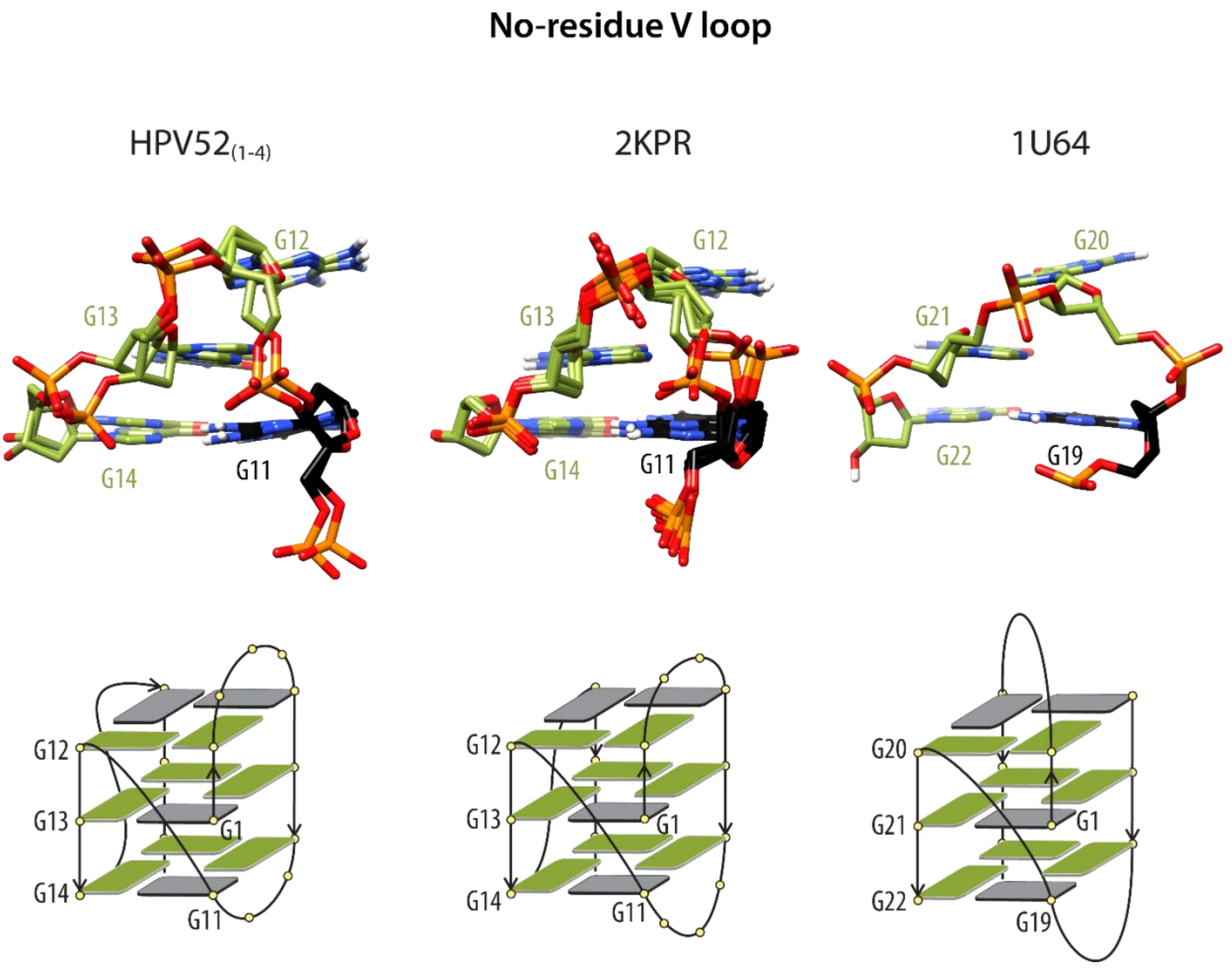

Figure S6: Comparison of a no-residue V loops in HPV52$_{(1\text{-}4)}$, *chl1* (pdb code 2KPR) and $(G_3T_3G_4)_2$ (pdb code 1U64) G-quadruplexes. Structural details of the loop and topology are shown for each structure. All different conformations with regard to the backbone angles observed in the structures are displayed.

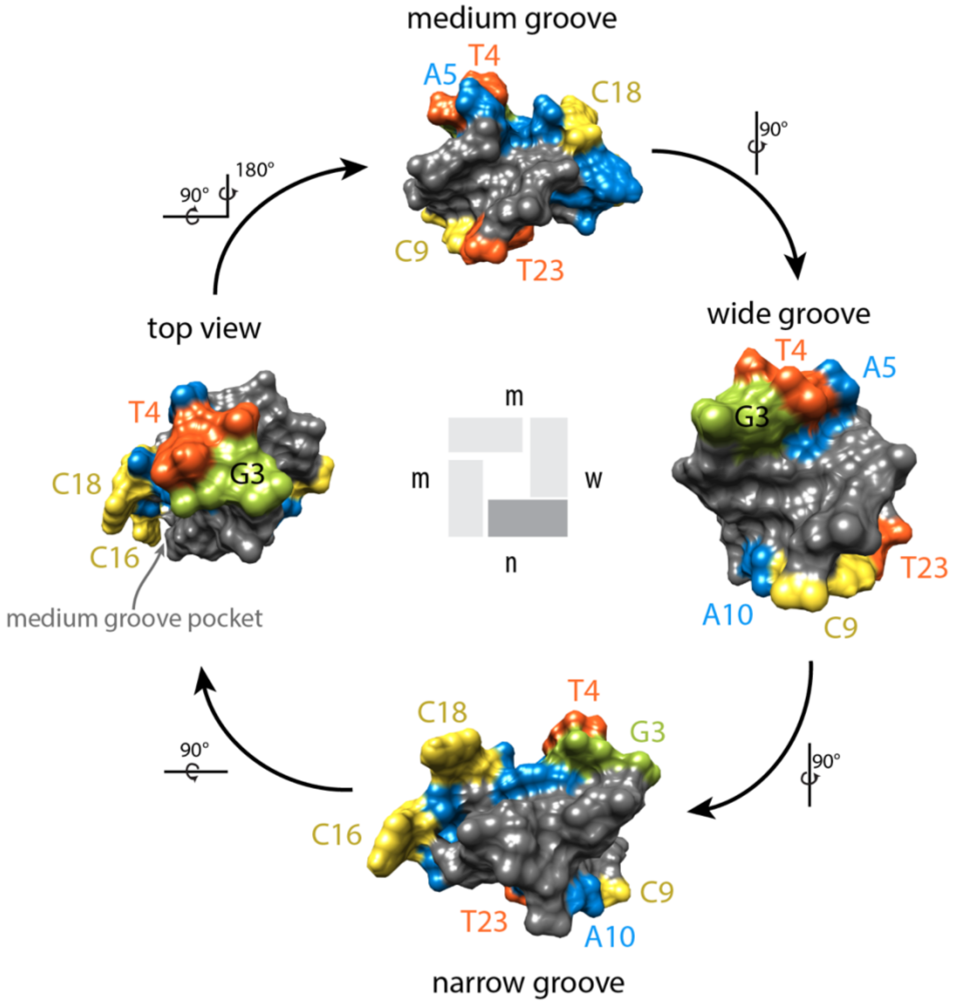

Figure S7: Surface representation of grooves of HPV52$_{(1-4)}$. Medium (top) and wide (right) grooves are completely accessible for hydrogen bond recognition of the G-quartet edges. Narrow groove (bottom) that accommodates a no-residue V-loop is inaccessible, while A15-C16-A17-C18-A19 propeller loop defines a pocket in medium groove between G12-G14 and G20-G22 tracts (left). G-quadruplex core, adenine, cytosine, guanine and thymine residues in lop are coloured grey, blue, yellow, green and red, respectively.
